# Novel gene mutation in von Hippel-Lindau disease – a report of two cases

**DOI:** 10.1186/s12881-019-0930-8

**Published:** 2019-12-10

**Authors:** Jitian Wang, Wenjie Cao, Zhaoxia Wang, Hong Zhu

**Affiliations:** Department of Cancer Medicine, Gaomi People’s Hospital, Shandong, 261500 Shandong Province China

**Keywords:** von Hippel-Lindau syndrome, von Hippel-Lindau gene, Novel mutation

## Abstract

**Background:**

Von Hippel-Lindau (VHL) syndrome is a familial autosomal dominant hereditary neoplastic disease caused by mutations in the VHL gene. Approximately 503 kinds of VHL gene mutations have been reported. Different types of mutations manifest various clinical phenotypes, from benign to malignant tumours or coexisting cysts. Thus, a gene mutation test is essential in the diagnosis of VHL syndrome.

**Case presentation:**

We reported two cases in which a novel mutation site in the c530-536delGACTGGA region in exon 3 of the VHL gene resulted in the development of VHL syndrome. According to the ACMG guidelines, this variation is pathogenic and consistent with autosomal dominant inheritance. This variation has not been reported anywhere in the databases or literature.

**Conclusion:**

This report will add a new mutation site to VHL gene databases. The newly added gene mutation and its associated clinical phenotypes will help improve the accuracy of VHL diagnosis and benefit the community of VHL gene mutation carriers.

## Background

VHL syndrome is an autosomal dominant hereditary neoplastic disease and is clinically characterized by hemangioblastoma, retinoblastoma, clear cell kidney carcinoma, pheochromocytoma and other cysts or tumours of the liver, kidney, pancreas and epididymis. Based on the presence or absence of pheochromocytoma, VHL syndrome can be classified as type 1 or type 2 [1, 2]. The average onset time of VHL syndrome in patients is approximately 26 years old, and VHL syndrome reaches a high penetrance of over 90% at the age of 65 years old [3]. With advances in genetic testing, gene mutations have become the standard in the diagnosis of VHL syndrome. Albiges and colleagues [4] have emphasized the importance of genetic testing in the diagnosis of VHL disease, particularly in patients with symptoms that are implicit or incompletely defined and in patients without a clear family history. Here, we reported 2 cases in which patients had clinical phenotypes like those of VHL syndrome, which was further confirmed by finding a novel mutation site in the VHL gene of the patients via gene sequence analysis. Thus, genetic testing will improve VHL diagnostic accuracy and has a significant advantage over the diagnosis based on clinical phenotypes alone.

## Case presentation

### Case 1

**Fig. 1:**
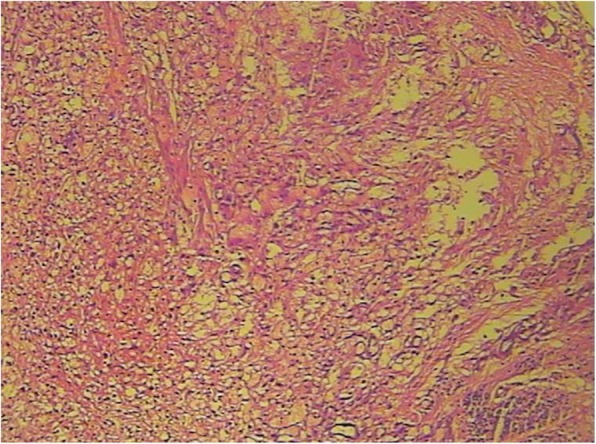
Cerebellar hemangioblastoma. Tissue was collected from the subject of case 1 and then formalin-fixed, paraffin-embedded sectioned and stained with haematoxylin-eosin. Images were taken under a regular microscope with a 10 X 10 lens

**Fig. 2:**
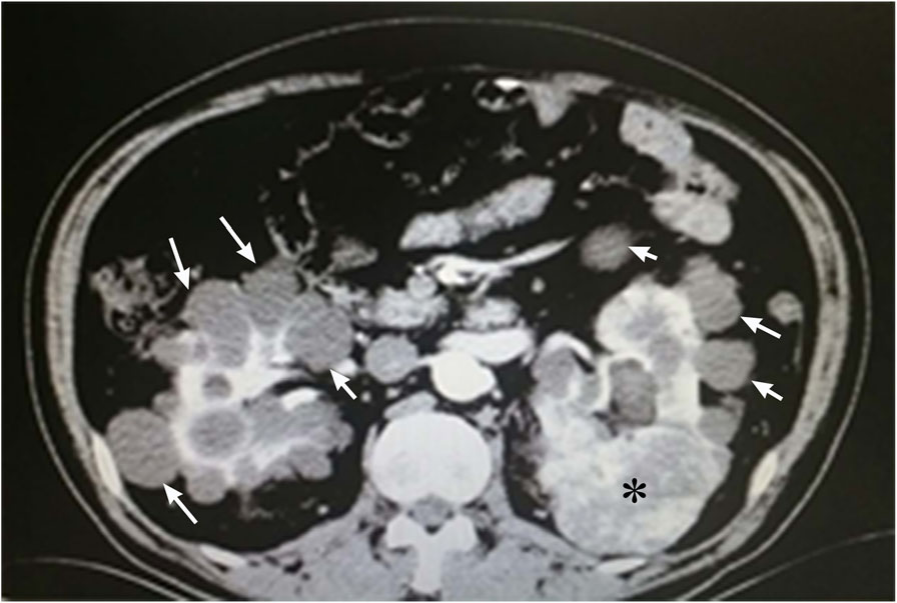
CT image of bilateral kidney tumours and polycystic kidneys. CT images were taken from both kidneys at the lumbar level from the subject of case 1. Arrows indicate multiple kidney cysts. Asterisks indicate kidney tumours

**Fig. 3:**
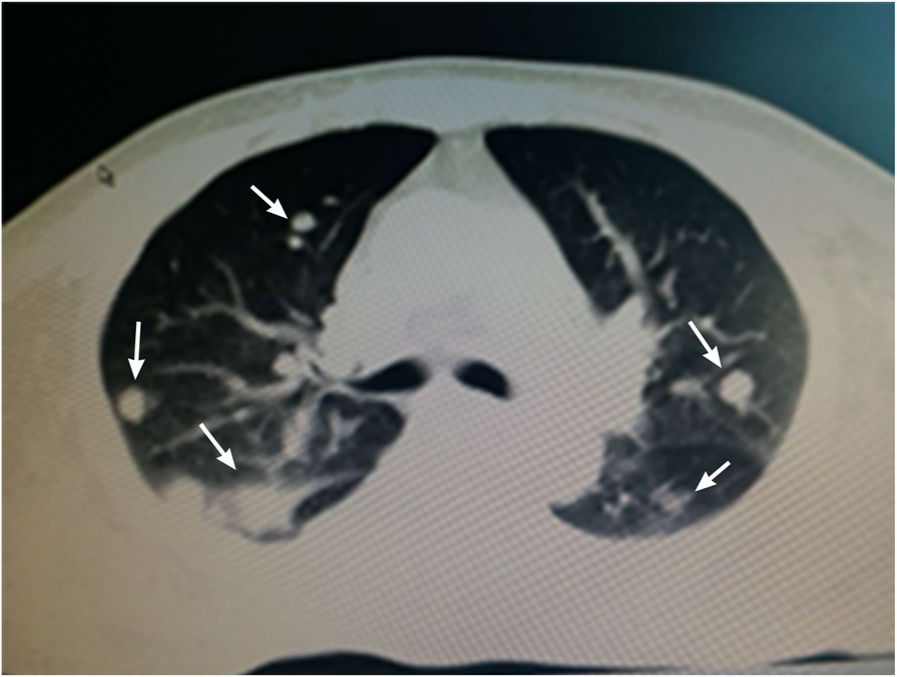
CT image of bilateral lung metastasis of tumours. A CT image was taken cross both lungs at the thoracic level from the subject of case 1. Arrows indicate multiple metastases of tumours to both sides of the lungs

This subject is a male, 54 years old, who was hospitalized in 2009 due to a headache that was diagnosed as a space-occupying lesion of the cerebellum via MRI examination. He was treated with a ventriculoperitoneal shunt and postoperative radiotherapy. In February 2011, he enrolled in our hospital due to dysfunction of the lower extremities. Contrast-enhanced MRI examination showed two large cysts in the bilateral cerebellum. Visible nodules with enhanced signals were found in the one cyst with a smaller size. Additionally, multiple nodular abnormalities were also found in the left cerebellum. Adjacent brain sulci were changed or disappeared due to enhanced pressure. The fourth ventricle showed unclear borders from the surrounding structures. Cerebellar tumour resection was performed, and pathologic analysis showed hemangioblastoma in the cerebellum (Fig. 1). On October 30, 2014, an enhanced CT examination showed that multiple renal cysts occurred in both kidneys with unclear borders, and the arterial phase was significantly enhanced with uneven density. The largest cyst was located in the left kidney, and its size was approximately 5.8 cm × 7.9 cm. Both kidneys showed low signal density due to multiple cystic lesions but partial calcification (Fig. 2). Multiple round water-like density shadows were observed in liver cirrhosis, with sharp edges but no enhancements. Considering the complications of kidney cancer, polycystic kidney disease and multiple liver cysts, the subject did not undergo further examination but underwent symptomatic treatments after discharge. In August 2017, the subject was enrolled in our hospital again due to dizziness and severe weight loss. CT examination of the cranial and thoracoabdominal regions showed multiple lacunar infarctions in the basal ganglia, bilateral softening lesions in the cerebellar hemispheres, multiple space-occupying lesions in both kidneys, bilateral polycystic kidney disease, multiple hepatic cysts and pulmonary nodules (Fig. 3). The patient’s father was dead, the cause of death was unknown, his mother was alive, and the patient was married and had a daughter.

**Fig. 4:**
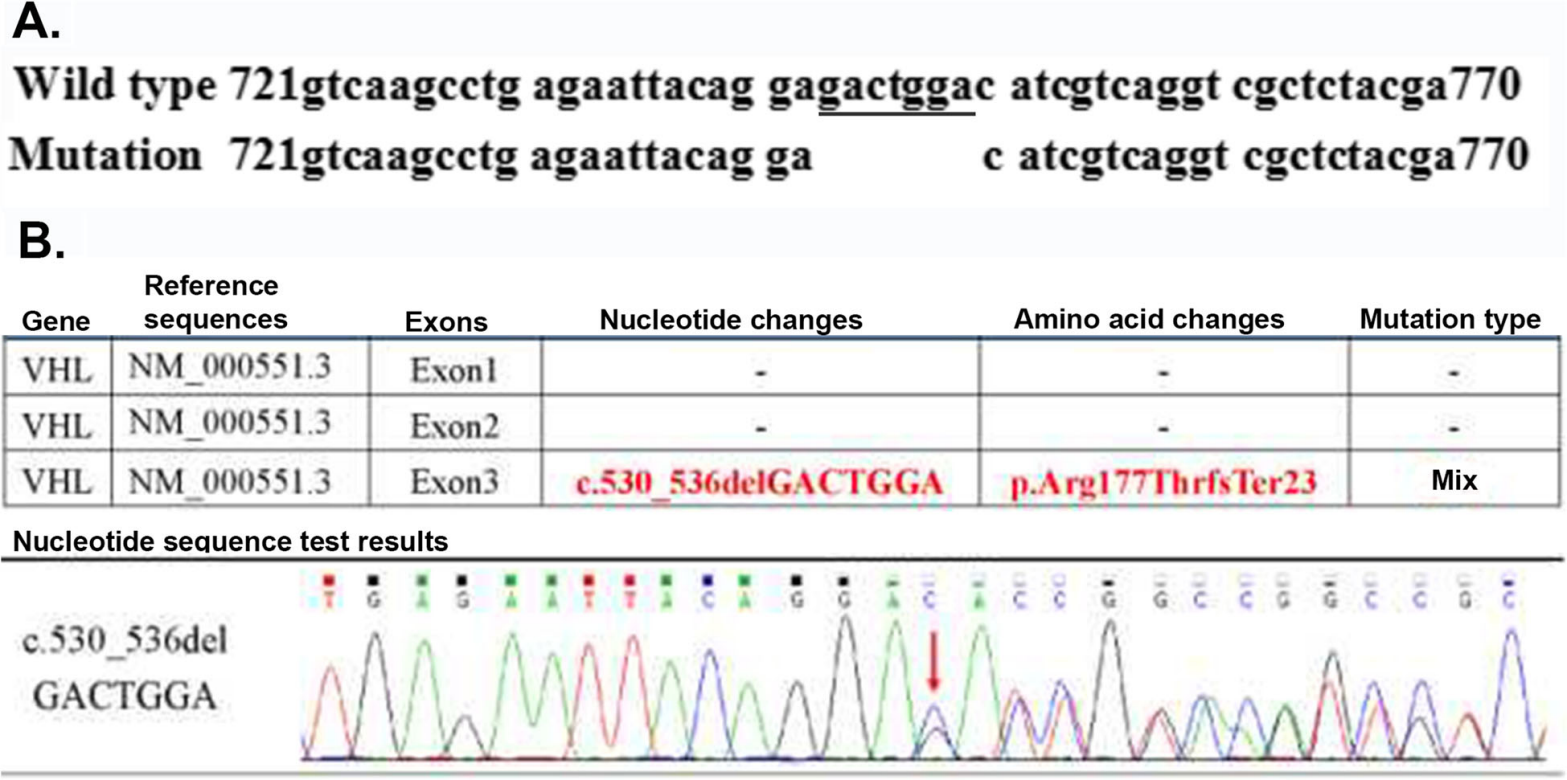
VHL gene and testing results of case 1. **a** The top row shows the wild-type human VHL DNA sequence segment of 721–770 from the NCBI database (NM_000551.3). The nucleotide database includes EST and GSS sequences. The bottom row shows the deletion of the GACTGGA sequence in the VHL gene of the subject. **b** Genetic testing results of the patient’s VHL gene, indicating the deletion of the GACTGGA sequence, leading to an amino acid Arg-177 change in case 1

**Fig. 5:**
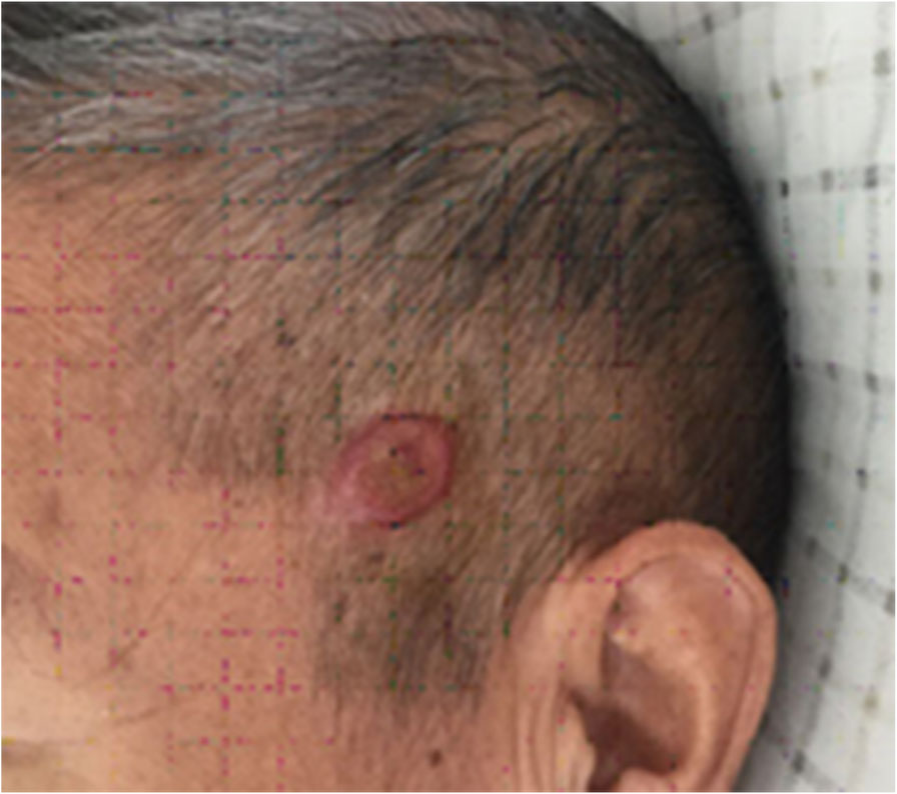
Cyst adenoma occurred on the head skin of the patient in case 1. This finding added phenotypic expression data to VHL variants

Physical examination: blood pressure 116/79 mmHg, consciousness, apparent anaemia, speech dysfunction. The left side of the temporal region had a visible mass with a size of approximately 0.5 cm × 2 cm × 3 cm that was red, hard, and fixed, with surface ulceration and no pus. The left occipital had old surgical scars. Limb muscle strength was at level 4. Laboratory tests: haemoglobin 81 g/L, glutamyl transpeptidase 352 U/L, albumin 30.6 g/L, serum sodium 128 mM, and creatinine 91 mol/L. The levels of tumour biomarkers were in the normal range. The patient was initially diagnosed with renal carcinoma that metastasized to the lungs. The patient and his relatives did not agree to perform further pathological diagnosis by renal biopsy. After a comprehensive analysis, it was thought that the patient might have VHL syndrome. Genomic DNA was extracted from the patient’s peripheral blood leukocytes using a DNA extraction kit, and VHL gene coding sequence analysis was performed by direct sequencing of PCR-amplified products using Sanger sequencing technology with an Applied Biosystems 3500 Dx Series Genetic Analyzer (ThermoFisher Scientific, USA) at Beijing Shengguzhi Medical Laboratory (Beijing, China) following the manufacturer’s instructions. It was found that a deletion mutation (Fig. 4) occurred in the patient’s VHL gene at the c530-536delGACTGGA region in exon 3. This deletion caused a change in the amino acid at position 177 (Arg-177, Fig. 4). Based on the results of genetic tests, the patient was diagnosed with VHL syndrome with renal carcinoma, bilateral lung metastases, multiple nephrotic cysts and multiple cysts of the liver, cerebellar hemangioblastoma and cystadenoma of the head (Fig. 5). The patient’s conditions were informed to his family members, and a family medical census was proposed but was ultimately declined. The application of vascular endothelial growth factor inhibitors to treat the disease was also refused. Other symptomatic and supportive treatments for anaemia and hypoproteinaemia were also declined. On August 26, 2017, CT examinations showed that the renal tumours and lung metastatic lesions increased compared with previous conditions. The patient discontinued treatments and died in 2018.

### Case 2

**Fig. 6:**
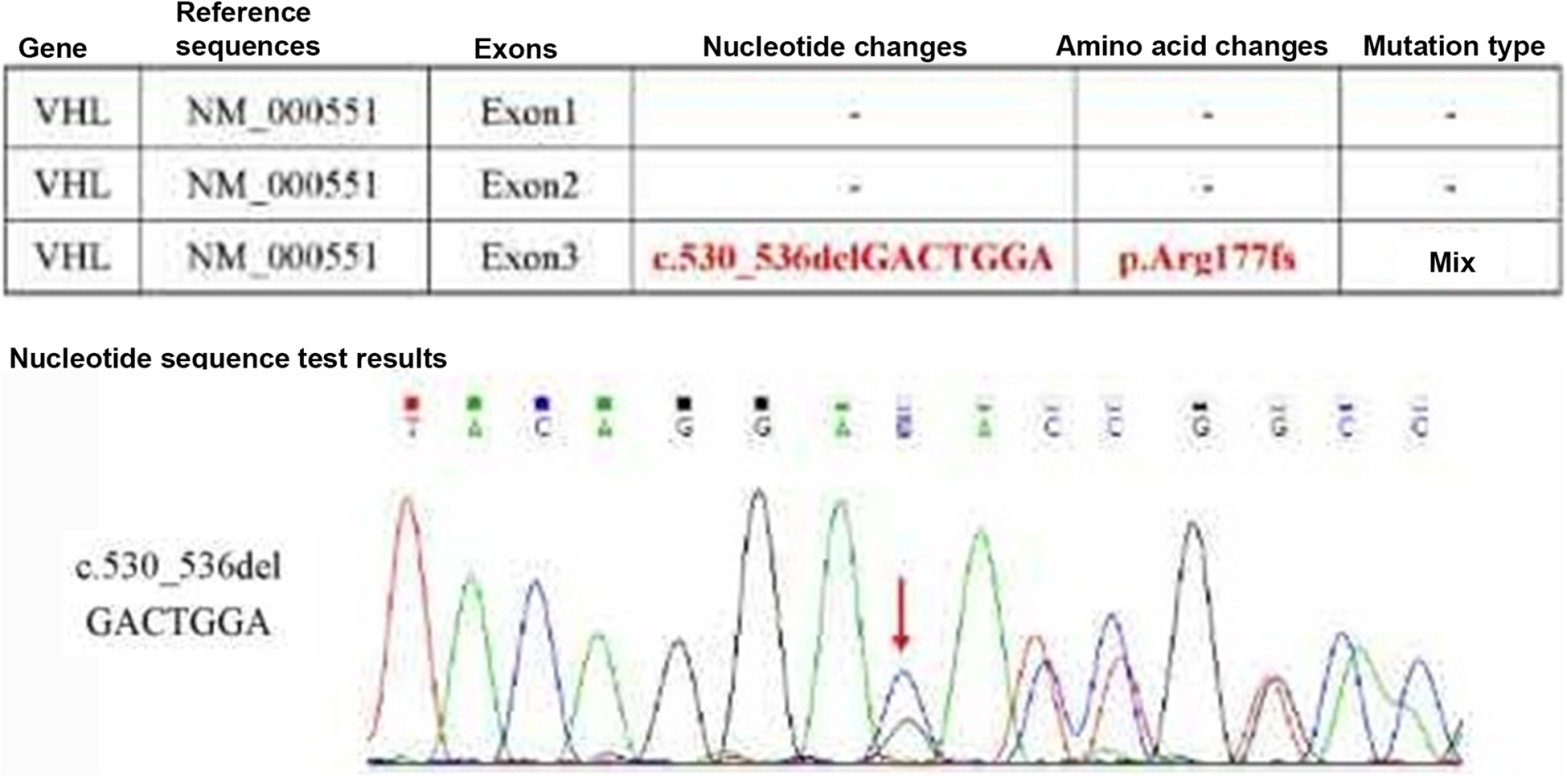
VHL gene testing results of case 2. The GACTGGA sequence was deleted in the VHL gene in case 2 by using extracted DNA from patient blood. This deletion resulted in the alteration of Arg-177 in the VHL protein

This subject is a female, 31 years old, who is the daughter of the subject in case 1 and was diagnosed as “seronegative arthritis” at the age of 12 along with a sympathetic polyarticular pain of the extremities. At 29-years-old, she was diagnosed with ankylosing spondylitis. The symptomatic treatment was ineffective. Deformation of joints was found in the extremities. She is currently unable to walk independently. In 2018, CT scans revealed multiple cysts in her kidney and pancreas but no abnormalities in her brain. VHL gene examination showed 1 missing variation (Fig. 6). The mutation site was the same as that in her father. She was diagnosed with VHL syndrome based on the results of genetic testing and her family history.

## Discussion and conclusion

VHL syndrome is a rare clinical disease, and its incidence is approximately 1:36000 [5]. Different types of mutations manifest various clinical phenotypes, from benign to malignant tumours or coexisting cysts. With limited clinical information in rural local hospitals in China, the diagnostic rate of VHL syndrome at an early stage is very low. Thus, once a subject is suspected to have VHL syndrome, VHL genetic testing should be performed as soon as possible to confirm the disease, rather than relying on the patient’s family history or basic physical and medical examinations alone.

The VHL gene is located on chromosome 3p25 ~ 26 and includes three exons. The loss of the gene, mutation and methylation inactivation are important molecular factors that lead to VHL disease. At least 503 kinds of VHL gene mutations have been found in VHL syndrome. Deletions, cleavage site mutations, nonsense mutations, missense mutations and frameshift mutations are common and account for 1732 entries of mutations in VHL syndrome in the COSMIC database. The three regions with more susceptible mutations are two cleavage sites between exons 2 and 3 at 75–82 and 157–189 and a hot mutation spot at codon 167. It was suggested by Zhang et al. [6] that the VHL gene missense mutation (Asn78Ser) was also has a high incidence. Gallou et al. [7] studied gene mutations in 173 primary sporadic human renal cell carcinomas, which were associated with mutations in the VHL tumour suppressor gene. They detected abnormal SSCP patterns in 73 samples. After sequencing, they identified microdeletions in 58% of cases, microinsertions in 17%, nonsense mutations in 8%, and missense mutations in 17%. Nordstrom-O ‘Brien et al. [3] studied the gene mutation types of 945 VHL pedigrees and found that multiple nonsense and frameshift mutations occurred in type 1 VHL syndrome. Point mutations usually occurred in codons 65, 76, 78 and 98, and cleavage site mutations occurred more often in codons 155, 158, 161, 162 and 167. Siu et al. [8] studied 5 families of type 1 VHL and 4 families of type 2 VHL mutations in the Chinese pedigree and found that mutations in type 2 VHL patients were only missense mutations and that patients with type 1 VHL had gene deletions, cleavage site mutations and nonsense mutations. Gossage et al. [9] suggested that the main reason for the onset of VHL in family members was based on a series of deletion mutations in the alleles of the wild-type VHL gene. Zhou et al. [10] reported that the mutation types and mutation sites of VHL syndrome were not uniform. It was difficult to determine where a hot mutation spot was located. VHL gene mutations could lead to a variety of organ tumours in various ways. The onset of tumour occurrence and tumour development was very difficult to predict, and its underlying mechanism has not yet been fully understood. Thus, studying the mutations of VHL syndrome by detecting and analysing VHL gene mutation sites will help us understand the pathogenesis of VHL syndrome and improve the diagnosis and treatments of VHL syndrome.

In the present cases, the whole sequence of the coding region of the VHL gene was analysed by PCR and Sanger sequencing. One deletion mutation was found at nucleotides 530–536 of exon 3 of the VHL gene. Seven bases were deleted (c.530_536delGACTGGA). This mutation resulted in alterations in the amino acid at position 177 (arginine) and the subsequent amino acids and led to the formation of an abnormal VHL protein. In a recent analysis [11] of the crystal contact of the VHL interface in Complex 2 to Brd4BD2, Arg-177 from VHL in chain D of Complex 1 interacts with Glu383 from Brd4BD2 in chain E of Complex 2, resulting in two observable conformations of the side chain and displacing VHL residue Arg-107 in Complex 2 (chain H) from the interface. This result indicates that Arg-177 plays an important role in VHL protein function. Indeed, the loss of Arg-177 and alterations in subsequent amino acids will reduce ubiquitin ligase E3 activity, leading to inefficiency in the ubiquitination and degradation of hypoxia-inducible factor (HIF). HIF is a transcription factor that plays a central role in the regulation of gene expression by oxygen. This novel mutation site in the VHL gene has not been reported in the HGMD, ClinVar, COSMIC and NCBI databases or in the literature [12, 13]. According to the ACMG guidelines [14, 15], this variation (c.530_536delGACTGGA in VHL) is pathogenic and consistent with autosomal dominant inheritance, as shown in the COSMIC database that the alteration of Arg-177 in VHL protein leads to VHL syndrome. Pathological examination showed hemangioblastoma in the cerebellar tumours of the case 1 patient. CT examination showed metastasis of renal carcinoma and bilateral multiple kidney cysts. Genetic testing revealed a novel pathogenic mutation in the VHL gene in both cases. Overall, these data met the clinical diagnostic criteria of VHL syndrome type 1 associated with deletion mutations. This finding will definitely add new information to databases of VHL gene mutations, add phenotypic expressions of the VHL mutations and improve the diagnostic accuracy of VHL disease, further benefitting VHL patients.

## Data Availability

The data used and/or analysed in the present report were deposited in the ClinVar database. The data are accessible via the accession number: SCV000963100; or via the links: https://www.ncbi.nlm.nih.gov/clinvar/variation/694389/; https://www.ncbi.nlm.nih.gov/clinvar/submitters/507133/

## Abbreviations

ACMG: American College of Medical Genetics and Genomics
CT: Computed tomography
MRI: Magnetic resonance imaging
VHL: Von Hippel-Lindau
